# Influenza vaccination coverage and effectiveness in young children in Thailand, 2011–2013

**DOI:** 10.1111/irv.12302

**Published:** 2015-01-05

**Authors:** Wanitchaya Kittikraisak, Piyarat Suntarattiwong, Jens Levy, Stefan Fernandez, Fatimah S Dawood, Sonja J Olsen, Tawee Chotpitayasunondh

**Affiliations:** a Influenza Program, Thailand Ministry of Public Health – U.S. Centers for Disease Control and Prevention CollaborationNonthaburi, Thailand; b Queen Sirikit National Institute of Child Health, Ministry of Public HealthBangkok, Thailand; c Armed Forces Research Institute of Medical SciencesBangkok, Thailand; d Influenza Division, U.S. Centers for Disease Control and PreventionAtlanta, GA, USA

**Keywords:** Influenza, vaccination, coverage, effectiveness, Thailand, children

## Abstract

**Background:**

Since 2009, Thailand has recommended influenza vaccine for children aged 6 months through 2 years, but no estimates of influenza vaccine coverage or effectiveness are available for this target group.

**Methods:**

During August 2011–May 2013, high-risk and healthy children aged ≤36 months were enrolled in a 2-year prospective cohort study. Parents were contacted weekly about acute respiratory illness (ARI) in their child. Ill children had combined nasal and throat swabs tested for influenza viruses by real-time reverse transcription–polymerase chain reaction. Influenza vaccination status was verified with vaccination cards. The Cox proportional hazards approach was used to estimate hazard ratios. Vaccine effectiveness (VE) was estimated as 100% x (1-hazard ratio).

**Results:**

During 2011–2013, 968 children were enrolled (median age, 10·3 months); 948 (97·9%) had a vaccination record and were included. Of these, 394 (41·6%) had ≥1 medical conditions. Vaccination coverage for the 2011–2012 and 2012–2013 seasons was 29·3% (93/317) and 30·0% (197/656), respectively. In 2011–2012, there were 213 ARI episodes, of which 10 (4·6%) were influenza positive (2·3 per 1000 vaccinated and 3·8 per 1000 unvaccinated child-weeks). The VE was 55% (95% confidence interval [CI], −72, 88). In 2012–2013, there were 846 ARIs, of which 52 (6·2%) were influenza positive (1·8 per 1000 vaccinated and 4·5 per 1000 unvaccinated child-weeks). The VE was 64% (CI, 13%, 85%).

**Conclusion:**

Influenza vaccination coverage among young children in Thailand was low, although vaccination was moderately effective. Continued efforts are needed to increase influenza vaccination coverage and evaluate VE among young children in Thailand.

## Background

Influenza is a serious global public health problem that accounts for 3–5 million severe illnesses and an average of 250 000–300 000 deaths worldwide each year.^1^ Certain underlying comorbidities confer a higher risk of severe influenza.^2^–^5^ In addition, children aged <60 months are at increased risk of severe influenza and influenza-associated hospitalization.^6^–^8^ Influenza vaccination is the most effective method for preventing influenza,^9^,^10^ and many countries

now have a seasonal influenza vaccination program targeting high-risk populations. The World Health Organization recommends seasonal influenza vaccination for pregnant women, healthcare workers, children aged 6–59 months, the elderly, and those with high-risk conditions.^11^

In Thailand, the burden of influenza among young children is high.^12^–^14^ In a case series of children aged <60 months, 10% of outpatient visits for influenza-like illness during 2005– 2006^12^ and 8% of hospitalizations for acute lower respiratory tract infections during 2005–2010 were associated with laboratory-confirmed influenza.^15^ In two rural Thai provinces, the estimated average annual incidence of hospitalized influenza among children aged <60 months was 236 per 100 000 population in 2005–2008 and 477 per 100 000 population during the 2009 H1N1 pandemic.^13^,^14^

Thailand recommends vaccination for persons aged ≥65 years, persons with underlying medical conditions, children aged 6 months through 2 years (<36 months), pregnant women, mentally ill persons, persons weighing >100 kg, and healthcare personnel. The availability of influenza vaccine in the public sector has been increasing, and in 2011, over seven million doses were sold (population 66 million people).^16^ At the national level, the Universal Coverage Scheme, a government-funded program, purchases influenza vaccine and distributes it to the Thai provinces. Vaccine is provided free of charge through public hospitals to target groups with priority given to persons ≥65 years and persons with underlying medical conditions. Vaccine coverage is thought to be very low (about 2% in young children).^17^

Using data from an ongoing cohort study of acute respiratory illness (ARI) among children in Bangkok, we evaluated influenza vaccination coverage and effectiveness among children in our cohort. This study was part of the larger study to measure rate of influenza acquisition and duration of influenza illness in healthy children and children with underlying medical conditions.

## Methods

### Study setting and population

In August 2011, we began an observational, prospective cohort study of children at the Queen Sirikit National Institute of Child Health (QSNICH), the largest public tertiary care hospital for children, in Bangkok, Thailand. Children were eligible for enrollment if they were residents of metropolitan Bangkok, 0–36 months of age, routinely sought care at QSNICH, and not acutely ill at enrollment. We aimed to enroll an equal number of healthy children and children with underlying comorbidities (prematurity/low birth weight [<37 weeks gestation or <2500 gram], congenital heart disease, chronic respiratory disease, immunosuppression, neurologic/neuromuscular conditions, and metabolic abnormalities). High-risk children were identified and approached when seeking care at clinics. An age- and time-matched child without any underlying comorbidities was then subsequently enrolled from the neonatal ward, well-baby clinic, or other outpatient clinics. The sequence of enrollment could also be reversed if a healthy child was first identified. Children who were already enrolled were followed through in the study even if their matches could not be found. After the consent process for which study nurses explained in detail about the study but did not discuss about specific respiratory disease prevention including vaccination, written informed consent was obtained from a parent or guardian of each child. This included consent to allow researchers to review children’s medical records to verify influenza vaccination status. Children were followed for 2 years. This study was approved by the ethics committee of the QSNICH.

### Data collection and laboratory studies

At enrollment, a trained study nurse administered a standardized questionnaire to each child’s caretaker to obtain baseline demographic and clinical data. Within 2 months of enrollment, the nurse visited the child’s home and conducted a survey to record family size, structure, and socioeconomic data. Each child’s vaccination card was reviewed at this home visit and again every 6 months. Study nurses called households weekly to assess whether the enrolled children had been ill with any new episode of ARI within the preceding 7 days. An ARI episode was defined as ≥2 of the following: fever, cough, sore throat, and runny nose. A new episode required ≥14 days of no symptoms between illnesses. For new episodes, the caretakers were asked to bring the children to the clinic to be evaluated, complete a brief clinical questionnaire, and have combined nasal and throat swabs collected. The caretakers were asked to contact the research team if the children developed symptoms requiring a clinic visit prior to weekly calls. Specimens were tested for influenza viruses by real-time reverse transcription–polymerase chain reaction (rRT– PCR).^18^ All children were treated by an attending physician according to Thailand’s standard of care. Study nurses called the caretakers 1–2 weeks later for a follow-up questionnaire of disease outcome.

### Influenza vaccination status and season

Because influenza viruses circulate year-round with a peak during June–October,^19^ we defined influenza season as June through May of the following year (e.g., June 2012–May 2013). Full vaccination was defined as having received two vaccine doses administered ≥28 days apart in the current season or two doses administered ≥28 days apart in any previous season and one dose in the current season.^20^ Partial vaccination was defined as having received one dose in the current season and never being fully vaccinated in any previous season. A child was classified as being unvaccinated if they were not vaccinated in the given season or if they received the first of two recommended influenza vaccine doses within 14 days before ARI onset during the given season. While we were able to collect data on vaccination status up to a year before study enrollment, ARI onset data, used to calculate vaccine effectiveness (VE), were only available from the start of the study (August 2011) onward. During the two seasons of this study, six children received two doses of influenza vaccine <28 days apart (range, 25– 27 days); they were classified as partially vaccinated for the purpose of analysis.

All seasonal influenza vaccines licensed and used in Thailand were trivalent, inactivated vaccines. Although both Northern Hemisphere and Southern Hemisphere vaccines were available for use in Thailand, all government purchased vaccine (about half the supply) is Southern Hemisphere vaccine and the majority of the privately purchased vaccine is Southern Hemisphere vaccine.^16^ The Southern Hemisphere vaccines for the 2011–2012 and 2012–2013 seasons included the same influenza strains: an A/California/7/2009 (H1N1) pdm09-like virus, an A/Perth/16/2009 (H3N2)-like virus, and a B/Brisbane/60/2008-like virus.^21^ The 2011–2012 Northern Hemisphere vaccine was also the same; however, the 2012–2013 Northern Hemisphere vaccine changed the H3 and B components (A/Victoria/361/2011 (H3N2)-like virus and B/Wisconsin/1/2010-like virus).

### Data analysis

Descriptive analysis was conducted to examine baseline demographic, socioeconomic, and clinical characteristics. For vaccination coverage and VE estimation, only children aged ≥6 months at the beginning of each influenza season were included in the analysis. Vaccination coverage was calculated by season. Only children with an onset date for the ARI were included in the VE calculation. VE was estimated as 100% x (1-hazard ratio) using the Cox proportional hazards approach. We used the Schoenfeld global goodness-of-fit test to test proportional hazards assumption. Other covariates included age at ARI, underlying medical condition (present/absent), and daycare attendance. Fully, partially, and any vaccinated children were compared with unvaccinated children. An influenza case was defined as an ARI episode for which the specimens tested positive for an influenza virus by rRT–PCR.

We conducted several sensitivity analyses in which we (i) shortened the influenza season from 12 months to 6 months,(ii) excluded ARI episodes in which the child took oseltamivir prior to swab collection, (iii) excluded influenza A (H3N2) from the 2012–2013 dataset because a drifted H3N2 strain emerged in 2012 affecting vaccine match to the circulating strains, (iv) included children with the 1st two doses administered <28 days apart as fully vaccinated, (v) excluded ARI episodes with specimens collected >7 days after illness onset, and (vi) shortened the time between last vaccination and onset of illness from 14 to 7 days. In addition, we examined the effect of prior vaccination on subsequent VE and if the VE differed by age and time since vaccination.

We calculated an ad hoc sample size. To detect a VE of 60% with significant 95% confidence limits, a minimum of 25 influenza cases among vaccinated children and five cases among unvaccinated children would be needed given the current follow-up time for each group. We defined a two-sided *P*-value of ≤0[C1]05 as statistical significance. All analyses were performed using Stata software version 11.0 (StataCorp LP, College Station, TX, USA).

## Results

### Study population

From August 2011–May 2013, we enrolled 968 children aged 0–36 months (median age at enrollment, 10[C1]3 months; interquartile range [IQR], 4[C1]7–19[C1]7 months). Of those, 948 (97[C1]9%) had available demographic data and known influenza vaccination status (for those aged ≥6 months) and were included in the analytic dataset. The median duration of follow-up was 50 weeks (IQR, 22–75 weeks). From August 2011–May 2013, there were 2134 respiratory illness episodes and 1660 (77[C1]8%) met the ARI definition. Of the 1660 ARI episodes, 154 (9[C1]2%) episodes, occurring in 135 children, were in children treated with oseltamivir prior to swab collection (30 [19[C1]5%] of these had laboratory-confirmed influenza). Among the 948 children included in the analysis, 512 (54[C1]0%) were male and 394 (41[C1]6%) had ≥1 underlying medical conditions (Table 1). The most common medical condition was prematurity/low birth weight, accounting for 25[C1]7% of all enrolled children, followed by heart disease (11[C1]6%) and chronic respiratory disease (10[C1]0%). The mothers of 23 children (2[C1]4%) reported receiving influenza vaccine during pregnancy. Most of the caretakers (39[C1]3%) finished secondary school. Thirty percent of the families earned between 10 000 and 19 999 Thai Baht/month (average monthly household income of a Thai family in 2011 was 23 236 Baht^22^; 30 Thai Baht = 1 US dollar). Most families (49[C1]5%) had 2–4 members (average household size for a Thai family in 2010 was 3[C1]2).^22^

**Table 1:** Baseline demographic, clinical, and socioeconomic characteristics of children enrolled into a prospective cohort study in Bangkok, Thailand

		Children aged ≥6 months at the beginning of study season	
	All children included in the analytic dataset, *N* < 948 *n* (%)	2011–2012 season, *N* < 317 *n* (%)	2012 2013 season, *N* < 656 *n* (%)
Age at enrollment (months)				
0 to <12	502 (52.9)	13 (4.1)	227 (34.6)	
12 to <24	266 (28.1)	136 (42.9)	249 (38.0)	
24 to 36	180 (19.0)	168 (53.0)	180 (27.4)	
Age at the beginning of study season (months)				
6 to <12	n/a	112 (35.3)	160 (24.4)	
12 to <24		162 (51.1)	291 (44.3)	
24 to <36		43 (13.6)	162 (24.7)	
≥36		0 (0.0)	43 (6.5)	
Male	512 (54.0)	180 (56.8)	359 (54.7)	
Underlying comorbidities*				
None	554 (58.4)	197 (62.1)	395 (60.2)	
Any	394 (41.6)	120 (37.9)	261 (39.8)	
Prematurity	244 (25.7)	64 (20.2)	155 (23.6)
Heart disease	110 (11.6)	35 (11.0)	68 (10.4)
Respiratory disease	95 (10.0)	48 (15.1)	76 (11.6)	
Neurologic/neuromuscular disorder	29 (3.1)	12 (3.8)	20 (3.0)	
Developmental delay	73 (7.7)	29 (9.1)	53 (8.1)	
Others	54 (5.7)	16 (5.0)	42 (6.4)	
Influenza vaccination				
Full	n/a	93 (29.3)	197 (30.0)	
Partial		31 (9.8)	94 (14.3)	
Mother received	23 (2.4)	8 (2.5)	19 (2.9)	
influenza vaccine				
during pregnancy				
Highest education of caretaker				
Never attended school	21 (2.2)	3 (0.9)	13 (2.0)	
Primary school	179 (18.9)	68 (21.5)	31 (20.0)	
Secondary school	373 (39.3)	118 (37.2)	252 (38.4)	
Vocational school	150 (15.8)	53 (16.7)	95 (14.5)	
University	222 (23.4)	74 (23.3)	162 (24.7)	
Not known/not	3 (0.3)	1 (0.3)	3 (0.5)	
answered/missing				
Monthly household income (Thai Baht)**				
1–9999	104 (11.0)	32 (10.1)	71 (10.8)	
10 000–19 999	287 (30.3)	99 (31.2)	204 (31.1)	
20 000–29 999	209 (22.0)	63 (19.9)	137 (20.9)	
30 000–39 999	141 (14.9)	51 (16.1)	101 (15.4)	
40 000 or more	207 (21.8)	72 (22.7)	143 (21.8)	
Number of household members				
2–4	469 (49.5)	152 (47.9)	328 (50.0)	
5–8	407 (42.9)	135 (42.6)	286 (43.6)	
>8	72 (7.6)	30 (9.5)	42 (6.4)	
				
				

n/a, not applicable (i.e., age at the beginning of study season was not applied for the whole analytic dataset because not all children were eligible for vaccination for a particular season).

* Not mutually exclusive.

** 30 Thai Baht = 1 US dollar. Kittikraisak et al. 88

At the beginning of the 2011–2012 season, 317 children aged ≥6 months were eligible for influenza vaccination and were included in the analysis. In the subsequent season, 339 children were newly enrolled and eligible for vaccination, resulting in the analytic dataset having 656 children in the 2012–2013 season. Baseline characteristics of children by vaccination status are shown in Table S1. Characteristics statistically associated with vaccination status were further explored in the analysis examining association between influenza virus infection and vaccination status.

#### Influenza vaccination coverage

Vaccination coverage (full or partial) by season was 39[C1]1% (124/317) in 2011–2012 and 44[C1]3% (291/656) in 2012–2013 (*P*-value = 0[C1]12; Table 2); 66[C1]1% and 75[C1]9% of vaccine were given in the first 6 months between June and November in 2011 and 2012, respectively. Full vaccination coverage by season was 29[C1]3% (93/317) and 30[C1]0% (197/656), respectively (*P*-value = 0[C1]82). For children aged <36 months, one of the priority groups for influenza vaccination, full vacci-nation coverage in the 2011–2012 season was similar to the coverage in the 2012–2013 season (93/317 [29[C1]3%] versus 189/613 [30[C1]8%]; *P*-value = 0[C1]64). Full vaccination coverage was significantly lower in healthy than in high-risk children in the 2011–2012 season (49/197 [24[C1]9%] versus 44/120 [36[C1]7%]; *P*-value = 0[C1]03). In contrast, full coverage was significantly higher in healthy than in high-risk children in the 2012–2013 season (130/395 [32[C1]9%] versus 67/261 [25[C1]7%]; *P*-value = 0[C1]05).

**Table 2:** Influenza vaccination coverage among children aged 6–44 months participating in a prospective cohort study in Bangkok, Thailand

	Vaccination coverage							
	2011–2012 season *n* (%)				2012–2013 season *n* (%)			
	*N*	Full	Partial	Any	*N*	Full	Partial	Any
Total	317	93 (29.3)	31 (9.8)	124 (39.1)	656	197(30.0)	94 (14.3)	291 (44.3)
6 to <12 months	112	36 (32.1)	6 (5.3)	42 (37.5)	160	51 (31.9)	17 (10.6)	68 (42.5)
12 to <24 months	162	41 (25.3)	16 (9.9)	57 (35.2)	291	85 (29.2)	46 (15.8)	131 (45.0)
24 to <36 months	43	16 (37.2)	9 (20.9)	25 (58.1)	162	53 (32.7)	24 (14.8)	77 (47.5)
≥36 months	0 0	(0.0)	0 (0.0)	0(0.0)	43 8(18.6)	7(16.3)	15(34.9)	
Healthy	197 49	(24.9)*	19(9.6)	68(34.5)	395	130(32.9)**	46(11.6)	176(44.5)
6 to <12 months	71	16 (22.5)	2 (2.8)	18 (25.3)	96	38 (39.6)	8 (8.3)	46 (47.9)
12 to <24 months	100	24 (24.0)	11 (11.0)	35 (35.0)	173	51 (29.5)	23 (13.3)	74 (42.8)
24 to <36 months	26	9 (34.6)	6 (23.1)	15 (57.7)	100	37 (37.0)	12 (12.0)	49 (49.0)
≥36 months	0	0 (0.0)	0 (0.0)	0 (0.0)	26 4 (15.4)	3 (11.5)	7 (26.9)	
Underlying comorbidities	120	44 (36.7)*	12 (10.0)	56 (46.7)	261	67 (25.7)**	48 (18.4)	115 (44.1)
6 to <12 months	41	20 (48.8)***	4 (9.7)	24 (58.5)	64	13 (20.3)	9 (14.1)	22 (34.4)
12 to <24 months	62	17 (27.4)***	5 (8.1)	22 (35.5)	118	34 (28.8)	23 (19.5)	57 (48.3)
24 to <36 months	17	7 (41.2)	3 (17.6)	10 (58.8)	62	16(25.8)	12 (19.3)	28 (45.2)
≥36 months	0	0 (0.0)	0 (0.0)	0 (0.0)	17	4 (23.5)	4 (26.5)	8 (47.0)

* Comparing full vaccination between children with underlying comorbidities and healthy children in the 2011–2012 season, *P*-value = 0.03.

** Comparing full vaccination between children with underlying comorbidities and healthy children in the 2012–2013 season, *P*-value = 0.05.

*** Comparing full vaccination between children with underlying comorbidities aged 6 to <12 months and 12 to <24 months in the 2011–2012 season, *P*-value = 0.03.

#### Influenza vaccine effectiveness

**Figure 1:**
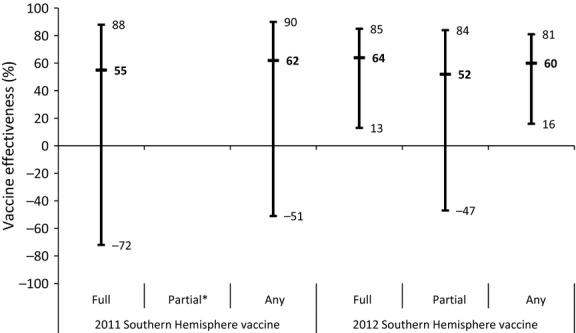
Effectiveness of the 2011 and 2012 Southern Hemisphere influenza vaccines in a prospective cohort of children in Bangkok, Thailand. Bold type indicates point estimate. Regular type indicates upper and lower limits of confidence interval. * Could not be calculated due to the small number of influenza cases

In the 2011–2012 season, there were 213 ARI episodes of which 10 (4[C1]7%), occurring in 10 children, were influenza positive (age at ARI range, 8[C1]9–36[C1]0 months). Six were positive for influenza A virus (H1N1pdm09 = 2 and H3N2 = 4) and four for influenza B virus. Zero of 14 specimens collected on the day of illness onset, 10 (5[C1]9%) of 169 collected 1–4 days after onset, zero of 20 collected 5– 7 days after onset, and zero of three collected >7 days after onset were positive for an influenza virus. The incidence of influenza virus infection among vaccinated and unvaccinated children was 2[C1]3 per 1000 child-weeks and 3[C1]8 per 1000 child-weeks, respectively (*P*-value = 0[C1]59). For 2011–2012, the VE for full vaccination adjusted for underlying medical condition was 55% (95% confidence interval [CI], [C0]72, 88; Figure 1). Further adjustment for age at ARI or daycare attendance did not meaningfully change the VE estimate. The VE for partial vaccination could not be calculated due to the small number of influenza cases. The VE for any vaccination was 62% (CI, [C0]51, 90).

There were 846 ARI episodes in the 2012–2013 season of which 52 (6[C1]1%) were influenza positive (age at ARI range, 10[C1]2–47[C1]7). Thirty were positive for influenza A virus (H1N1pdm09 = 15, H3N2 = 14, and not typed = 1) and 22 for influenza B virus. Seven (15[C1]5%) of 45 specimens collected on the day of illness onset, 43 (6[C1]1%) of 699 collected 1–4 days after illness onset, two (2[C1]4%) of 84 collected 5–7 days after onset, and zero of 11 collected >7 days after onset were positive for an influenza virus. Fifty children had one influenza-confirmed ARI during this season, while one child had two influenza-confirmed ARIs. The incidence of influenza virus infection among vaccinated children was 1[C1]8 per 1000 child-weeks, while that of unvaccinated children was 4[C1]5 per 1000 child-weeks (*P*-value <0[C1]01). The VE for full vaccination adjusted for underlying medical condition was 64% (CI, 13%, 85%; Figure 1). The VE further adjusted for age at ARI or daycare attendance remained unchanged. In the 2012–2013 season, the VE for partial and any vaccination was 52% (CI, [C0]47%, 84%) and 60% (CI, 16%, 81%), respectively. The age-specific VE for full vaccination among children aged 6 months through 2 years (<36 months) in the 2012–2013 season was 79% (CI, 29%, 94%; Table 3).

**Table 3:** Effectiveness of the 2011 and 2012 Southern Hemisphere influenza vaccines in various scenarios

		2011 Southern Hemisphere vaccine		2012 Southern Hemisphere vaccine	
Scenario	Vaccination status	Vaccine effectiveness	95% confidence interval	Vaccine effectiveness	95% confidence interval
Adjust for age at ARI (continuous variable) and the presence of underlying medical condition	Full	57	–46, 87	64	12, 85
Exclude ARI episodes occurring during the last 6 months of season	Full	55	–72, 88	64	13, 85
Exclude all ARI episodes in persons treated with oseltamivir before swab collection	Full	75	–53, 96	57	–31, 86
Exclude ARI episodes that tested positive for A(H3N2) in 2012–2013 season	Full	n/a	n/a	63	2, 86
Re-classify children with 1st 2 doses administered <28 days apart as fully vaccinated	Full	57	–67, 89	64	13, 85
Exclude episodes with specimens collected =7 days of illness onset	Full	78	–31, 96	61	50, 84
Shorten time between last vaccination and onset of illness from 14 to 7 days	Full	58	–64, 89	65	13, 86
Include only children aged <36 months	Full	46	–103, 86	79	29, 94
Include only children with ≤3 months between vaccination and ARI onset	Full	*	*	3	–154, 63
Include only children with =3–6 months between vaccination and ARI onset	Full	*	*	61	–12, 87
Include only children with =6–9 months between vaccination and ARI onset	Full	*	*	84	6, 97

ARI, acute respiratory illness; n/a, not applicable.

* Could not be calculated due to small sample size.

In general, the sensitivity analyses had little effect on the VE point estimates (Table 3). Among the 102 children with two complete years of vaccine records, there were only 27 influenza cases and the data were too sparse to examine the effect of prior vaccination on VE. Time since vaccination had some impact on VE estimates. In the 2012–2013 season, VE in the first 3 months since vaccination was 3% (CI, [C0]154%, 63%). The estimate increased to 61% (CI, [C0]12%, 87%) and 84% (CI, 6–97%) for the period of >3–6 months and >6–9 months, respectively. We did not have enough cases to examine the VE stratified by time since vaccination for the 2011–2012 season.

### Discussion

Influenza vaccination coverage was fairly low in this cohort of young children in Bangkok, possibly due to a relatively limited vaccine supply compared to the size of the population in vaccination target groups. Our estimate of influenza VE in the 2011–2012 season did not reach statistical significance due to the small number of influenza virus infections in the cohort. However, the point estimate of influenza VE suggests that the 2012 Southern Hemisphere vaccine was moderately effective (64%) at protecting young children against influenza and this increased to 79% among the target age-group; these data are consistent with influenza VE estimates among children in this age-group from other countries.^23^–^32^

In absolute terms, full vaccination coverage in our cohort of young children was low at 30%. However, our coverage estimates are much higher than preliminary estimates from the Universal Coverage Scheme during the same period (2[C1]3%).^17^ They are also higher than estimates from Canada (4–6% fully vaccinated during the 2006–2009 seasons) and are comparable to estimates after the first few years of influenza vaccination recommendations for children in the United States (4[C1]4% in 2002–2003, 8[C1]4% in 2003–2004, 17[C1]8% in 2004–2005, 20[C1]6% in 2005–2006, 21[C1]3% in 2006–2007, and 23[C1]4% in 2008–2009).^33^–^40^ Compared to the national estimates, coverage may have been higher in our cohort for several reasons, including increased access to vaccine through the private sector due to residence in a metropolitan area, increased awareness among caretakers because of the 2009 influenza pandemic, increased attention to preventing influenza in children among staff at the study site which is a tertiary care academic pediatric facility, closer residence to the study facility which facilitated easy access to medical care, and increased proportion of children with comorbidities who are a recommended priority group for influenza vaccination. Fifty percent of eligible unvaccinated children received the first dose of influenza vaccine after enrolling in the study and receiving regular follow-up at the study facility. Although the vaccination coverage findings from this study may not be generalizable to all Thai children, they highlight that influenza vaccination coverage among young children remains suboptimal, leaving much room for improvement. Furthermore, influenza vaccination in Thailand is considered optional in children unless they belong to one of the risk groups recommended for vaccination by the Ministry of Public Health, and because of limited vaccine supply (approximately 25% of all recommended individuals), vaccination coverage remains low.

In general, we found that medically high-risk and healthy children had different influenza vaccination coverage, and we observed some differences in their household and demographic characteristics. It is possible that these children were not drawn from the same population. Nonetheless, children in our cohort came from families comparable to other Thai families in terms of household income and number of household members.

The VE estimates for both years were consistent with the limited data from children <36 months of age reported elsewhere from earlier years. In other countries, the influenza VE in this population has ranged from 47 to 89%.^23^–^32^

Although globally there was concern that the match between circulating influenza A (H3N2) strains and that component of the vaccine in 2012 was poor^41^,^42^ (26% antigenic match in Thailand, M. Chittaganpitch, personal communication), exclusion of those episodes did not increase VE in our study. However, it may be that we only had a few ARI episodes associated with influenza A (H3N2) in our dataset.

Our study has several strengths and limitations. Ascertainment bias of influenza status was minimized using rRT– PCR, an assay that is maximally sensitive and specific for influenza viruses. Further, this study was conducted in a defined cohort with nearly 100% of ARI episodes managed at our study hospital, hence reducing potential for missed influenza cases. The bias from overreporting of children’s vaccination uptake by parents was minimized in our study by the review of vaccination card, a preferable method for determining vaccination status in young children.^43^ Nonetheless, children who got vaccinated may have been different from those who did not in ways that our study did not measure, and children in our cohort were not necessarily representative of those in greater Bangkok. Additionally, we calculated VE for Southern Hemisphere vaccines yet each season theoretically spanned both Southern Hemisphere and Northern Hemisphere vaccine distribution which could have attenuated our VE estimates. However, during this time, most vaccine in Thailand was Southern Hemisphere vaccine and our sensitivity analysis of the first 6 months of data did not change the results. Nevertheless, future VE studies in Thailand should ascertain which vaccine formulation was received to improve precision of the VE estimate. Finally, the study was not designed to estimate VE so was underpowered in the 2011–2014 season.

To our knowledge, this is the first study in Thailand to assess influenza vaccination coverage and effectiveness among young children. Influenza viruses frequently undergo antigenic drift necessitating annual reformulation of seasonal influenza vaccines. Hence, continued evaluation of influenza vaccination coverage and effectiveness is merited to evaluate national influenza vaccination program impact among young children who are at high risk of severe influenza.

## Supporting Information

**Table S1.** Baseline demographic characteristics of children enrolled into a prospective cohort study in Bangkok, Thailand by vaccination status.
