# Supplementary material for: Influenza vaccination coverage and effectiveness in young children in Thailand, 2011–2013
Source: Influenza Other Respir Viruses. 2015 Jan 5;9(2):85–93. doi: 10.1111/irv.12302 (PMC4353321; doi:10.1111/irv.12302)
Supplement: Supplementary file 1 [file irv0009-0085-sd1.doc]

**Table S1.** Baseline demographic characteristics of children enrolled into a prospective cohort study in Bangkok, Thailand by vaccination status

|  | 2011-2012 season | | | 2012-2013 season | | |
| --- | --- | --- | --- | --- | --- | --- |
|  | Unvaccinated, N=193  n (%) | Vaccinated, N=124  n (%) | p-value | Unvaccinated, N=365  n (%) | Vaccinated, N=291  n (%) | p-value |
| Age at the beginning of study season (months)  6 to <12  12 to <24  24 to <36  ≥36 | 70 (36.3)  105 (54.4)  18 (9.3)  0 (0.0) | 42 (33.9)  57 (46.0)  25 (20.2)  0 (0.0) | 0.02 | 92 (25.2)  160 (43.8)  85 (23.3)  28 (7.7) | 68 (23.4)  131 (45.0)  77 (26.5)  15 (5.1) | 0.47 |
| Male | 111 (57.5) | 69 (55.7) | 0.74 | 185 (50.7) | 174 (59.8) | 0.02 |
| Underlying co-morbidities* | 64 (33.2) | 56 (45.2) | 0.03 | 146 (40.0) | 115 (39.5) | 0.90 |
| Daycare attendance at enrollment | 33 (17.1) | 29 (23.4) | 0.17 | 53 (14.5) | 38 (13.1) | 0.59 |
